# Application of Hydrogen Peroxide to the Control of Eutrophic Lake Systems in Laboratory Assays

**DOI:** 10.3390/toxins6092657

**Published:** 2014-09-09

**Authors:** Letizia Bauzá, Anabella Aguilera, Ricardo Echenique, Darío Andrinolo, Leda Giannuzzi

**Affiliations:** 1 Cátedra de Toxicología, Facultad de Ciencias Exactas, Universidad Nacional de La Plata, 48 y 115, La Plata 1900, Argentina; E-Mails: letiziabau@yahoo.com.ar (L.B.); dandrinolo@yahoo.com (D.A.); 2 INBIOTEC-CONICET y CIB-FIBA, Vieytes 3103, Mar del Plata 7600, Argentina; E-Mail: anabella.aguilera@gmail.com; 3 División Ficología, Facultad de Ciencias Naturales y Museo, Universidad Nacional de La Plata, Paseo del Bosque s/n°, La Plata 1900, Argentina; E-Mail: rechen@fcnym.unlp.edu.ar

**Keywords:** cyanobacteria, coliforms, chemical oxygen demand, hydrogen peroxide, lake management, *Planktothrix agardhii*

## Abstract

We exposed water samples from a recreational lake dominated by the cyanobacterium *Planktothrix agardhii* to different concentrations of hydrogen peroxide (H_2_O_2_). An addition of 0.33 mg·L^−1^ of H_2_O_2_ was the lowest effective dose for the decay of chlorophyll-a concentration to half of the original in 14 h with light and 17 h in experiments without light. With 3.33 mg·L^−1^ of H_2_O_2_, the values of the chemical oxygen demand (COD) decreased to half at 36 and 126 h in experiments performed with and without light, respectively. With increasing H_2_O_2_, there is a decrease in the total and faecal coliform, and this effect was made more pronounced by light. Total and faecal coliform were inhibited completely 48 h after addition of 3.33 mg·L^−1^ H_2_O_2_. Although the densities of cyanobacterial cells exposed to H_2_O_2_ did not decrease, transmission electron microscope observation of the trichomes showed several stages of degeneration, and the cells were collapsed after 48 h of 3.33 mg·L^−1^ of H_2_O_2_ addition in the presence of light. Our results demonstrate that H_2_O_2_ could be potentially used in hypertrophic systems because it not only collapses cyanobacterial cells and coliform bacteria but may also reduce chlorophyll-a content and chemical oxygen demand.

## 1. Introduction

Cyanobacteria, also known as blue-green algae, are commonly observed in nutrient-rich aquatic ecosystems. Cyanobacteria can be found all over the world and often form dense blooms. Such blooms are problematic: the landscape deterioration is accompanied by a reduction in recreational value; the processing costs for water purification increase significantly and the decrease in water quality causes serious sanitary problems such as conditions that allow the settlement of disease vectors [1]. Under favorable light and nutrient conditions, some species produce toxic secondary metabolites known as cyanotoxins [2]. Cyanotoxins constitute a health-risk for human beings worldwide via recreational and drinking water and have been linked to the deaths of wild and domestic animals all over the world [3]. Evidence suggests that toxic cyanobacterial metabolites can bioaccumulate in aquatic food-webs, which can cause the additional health concern of food-borne contaminants [4].

The changes in temperature and precipitation that accompany global warning are predicted to stimulate increased cyanobacterial growth rates. The fourth assessment report of the Intergovernmental Panel on Climate Change [5] predicted more frequent toxic blooms. For this reason, controlling cyanobacterial blooms in recreational water bodies and cyanotoxin levels in tap water is essential to assure water quality.

Many methods of controlling cyanobacterial blooms have been studied, including the reduction of nutrient inputs, the mechanical removal of cyanobacterial biomass, artificial destratification, ultrasonication, bacterial and chemical degradation [6]. Several techniques are potentially harmful to the environment and destroy only cyanobacterial cells, leaving the toxins to be released into the surrounding water following cell lysis [7]. Conventional water treatment is usually not effective in removing extracellular cyanotoxins (soluble toxins). Neither aeration nor air stripping are effective treatments for removing soluble toxins or cyanobacterial cells [8]. Advanced treatment processes, such as oxidant agents, must be implemented to remove both extracellular toxins and intact cells.

Hydrogen peroxide is a well-known agent for disinfection and water treatment with a strong oxidizing capability. Its ability to inhibit coliform bacteria and remove COD could represent an important advance in methods of pre-treatment in drinking water plants. Barrington and Gadouani [9] proposed it as a cyanobacterial inhibitor compound for aquaculture and indicated that cyanobacteria were more sensitive to it than other photoautotrophs. H_2_O_2_ has also been demonstrated to be an effective chemical algaecide to inhibit cyanobacteria [10]. Because H_2_O_2_ is a strong oxidizing agent and environmentally friendly, it can be effective for removing COD and inhibiting the coliform bacteria in reservoirs used for drinking water.

The effect of light can enhance the toxicity of relatively high H_2_O_2_ concentrations in aquatic plants and phytoplankton [11]. In addition, hydrogen peroxide can destroy cyanobacteria and cyanotoxins [12,13], and it is well-established as an environmentally-friendly oxidizing agent because of its rapid decomposition into oxygen and water [14].

In this work, we used laboratory assays to study the effect of H_2_O_2_ on the parameters of eutrophication and on phytoplankton, with a focus on cyanobacteria. In addition, faecal coliform and COD were also analyzed. We used water samples taken from a shallow lake in the Province of Buenos Aires, Argentina, which has been dominated by cyanobacteria for more than ten years. Experiments were conducted with and without light in order to evaluate the effect of light on the effectiveness of H_2_O_2_.

## 2. Results and Discussion

### 2.1. Physicochemical and Biological Conditions of the Shallow Lake

Los Patos, a shallow lake, is highly turbid with dense phytoplankton. Its chemical parameters were measured immediately after arrival at the laboratory (time zero) and are shown in Table 1.

Phosphorus content is one of the factors most likely to limit cyanobacteria growth in water ecosystems. Lake Los Patos showed total and dissolved phosphorus values of 0.89 and 0.20 mg·L^−1^ respectively. When total phosphorus exceeds 0.10 mg·L^−1^, the lake is usually in a turbid-water stable state dominated by phytoplankton [15]. Similar levels of total phosphorus were reported by Ruiz [16] in the San Roque Dam, a hypertrophic water body placed in Cordoba, Argentina, where total phosphorus reached 0.886 mg·L^−1^.

High levels of chlorophyll-a (Chl-a) (530 μg·L^−1^) were also obtained. The concentration of Chl-a in water bodies provides a reasonable estimate of algal biomass. The international guidelines for safe practices in managing recreational waters [17] has linked both short-term adverse health outcomes, e.g., skin irritation and gastrointestinal illness, and the potential for long term illness to Chl-a concentrations above 50 μg·L^−1^. The Chl-a concentration registered in Los Patos lake exceeded this value more than tenfold.

COD is commonly used to represent the amount of organic materials that can be chemically oxidized, and was identified as a very significant environmental factor for algal growth in water bodies [18]. In this work, high levels of COD (243 mg·L^−1^) were obtained in Los Patos lake (Table 1).

The phytoplankton assemblage was mainly composed of cyanobacteria, which accounted for 99% of the total phytoplankton cell density (average density of 2.4 × 10^7^ cells mL^−1^). *Planktothrix agardhii* (1.7 × 10^7^cells mL^−1^) and *Aphanizomenon aphanizomenoides* (7.0 × 10^6^ cells mL^−1^) dominated among cyanobacteria, accounting for 70.8% and 29.1% of the total phytoplankton, respectively. Other cyanobacteria such as *Anabaenopsis aff. elenkinii*, *A. cunningtoni*, *Raphidiopsis mediterranea* and* Microcystis aeruginosa* were present in percentages less than 1% (4.0 × 10^3^ cells mL^−1^). Chlorophyta and Chrysophyta represented less than 1% of total phytoplankton with counts lower than 2.0 × 10^3^ cells mL^−1^.

According to Chapman [19], the values of total phosphorus (0.84 mg·L^−1^), Chl-a (530 μg·L^−1^) and total phytoplankton density (2.4 × 10^7^ cells mL^−1^) obtained in the Los Patos lake showed that this lake could be classified as hypertrophic, with elevated concentrations of nutrients and an associated high biomass production. Trophic State Indices (TSI-_t_P and TSI-Ch-a) were calculated following Equations (1) and (2) (see materials and methods section). Both TSI metrics (TSI-_t_P = 96.09, TSI-Ch-a = 93.05) indicated that the ecosystem is hypertrophic.

**Table 1:** Physicochemical characteristics of Los Patos shallow lake at the time zero and at after of 48 h of H_2_O_2_ addition.

Parameters	Time Zero	Light	48 h after H_2_O_2_ addition					
			0	0.17 (mg L^−1^)	0.33 (mg L^−1^)	0.83 (mg L^−1^)	1.67 (mg L^−1^)	3.33 (mg L^−1^)
Conductivity (μS cm^−1^)	599 ± 15	Yes	618 ± 12	666 ± 12	662 ± 20	665 ± 27	630 ± 19	640 ± 18
		No	680 ± 18	667 ± 19	670 ± 21	660 ± 25	630 ± 20	645 ± 20
pH	9.47 ± 0.20	Yes	8.88 ± 0.10	9.05 ± 0.13	9.00 ± 0.12	9.06 ± 0.09	9.19 ± 0.12	9.22 ± 0.12
		No	7.87 ± 0.12	7.45 ± 0.18	7.44 ± 0.10	7.68 ± 0.15	8.06 ± 0.15	8.20 ± 0.10
Optical Density	0.37 ± 0.02	Yes	0.08 ± 0.01	0.07 ± 0.01	0.09 ± 0.02	0.09 ± 0.01	0.08 ± 0.02	0.08 ± 0.02
		No	0.08 ± 0.02	0.07 ± 0.01	0.09 ± 0.02	0.09 ± 0.01	0.08 ± 0.01	0.07 ± 0.01
Total Phosphorus (mg L^−1^)	0.89 ± 0.10	Yes	0.87 ± 0.10	0.83 ± 0.12	0.82 ± 0.09	0.81 ± 0.12	0.87 ± 0.03	0.90 ± 0.08
		No	0.89 ± 0.09	0.79 ± 0.10	0.81 ± 0.12	0.96 ± 0.15	0.91 ± 0.06	0.94 ± 0.09
Dissolved total Phosphorus (mg L^−1^)	0.20 ± 0.08	Yes	0.18 ± 0.08	0.40 ± 0.10	0.40 ± 0.09	0.55 ± 0.13	0.53 ± 0.09	0.55 ± 0.09
		No	0.16 ± 0.08	0.30 ± 0.12	0.31 ± 0.13	0.47 ± 0.12	0.49 ± 0.10	0.53 ± 010
Chl-a (μg L^−1^)	530 ± 10	Yes	445 ± 12	117 ± 5	74 ± 3	77 ± 8	71 ± 9	58 ± 8
		No	420 ± 22	320 ± 9	75 ± 6	81 ± 8	117 ± 7	111 ± 5
COD (mgO_2_ L^−1^)	243 ± 12	Yes	212 ± 8	142 ± 6	129 ± 9	130 ± 9	120 ± 10	126 ± 5
		No	213 ± 10	226 ± 15	205 ± 11	200 ± 12	180 ± 9	119 ± 7
Total coliforms (MPN × 100 mL^−1^)	4600	Yes	4600	2800	2400	150	90	<3
		No	4600	2800	2400	280	93	<3
Fecal coliforms (MPN × 100 mL^−1^)	4600	Yes	4600	2100	2100	130	11	<3
		No	4600	2100	2100	210	14	<3

### 2.2. Effect of H_2_O_2_ on Total and Faecal Coliform Bacteria Counts

Both total and faecal coliforms were found in high numbers, up to 600 MPN × 100 mL^−1^ before treatment (Table 1). These values exceeded the upper limit for recreational water bodies by more than a factor of 20 (200 MPN × 100 mL^−1^ of faecal coliforms) [20].

With increasing H_2_O_2_, the MPN × 100 mL of total and faecal coliforms decreased, and this effect was more pronounced in light (Table 1). There was complete inhibition 48 h after 3.33 mg·L^−1^ H_2_O_2_ was added. These results indicate the effectiveness of the H_2_O_2_ at removing these types of bacteria.

The bactericidal efficacy of hydrogen peroxide has been demonstrated in both water and food systems [21,22,23], with gram negative organisms showing greater susceptibility [24,25]. This antimicrobial action stems from its ability to form reactive oxygen species such as the hydroxyl radical and singlet oxygen, which can damage DNA and membrane constituents [26].

### 2.3. Effect of H_2_O_2_ on Chemical Parameters

**Figure 1:**
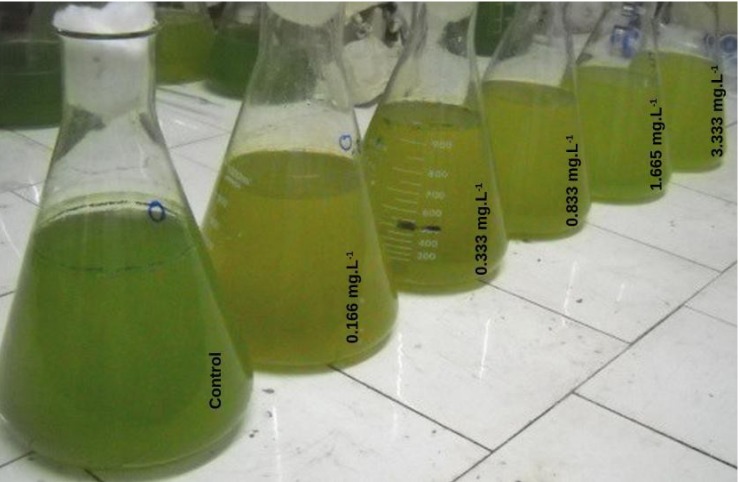
The effect of H_2_O_2_ addition on the appearance of water samples after 48 h after H_2_O_2_ in the presence of light.

The addition of H_2_O_2_ caused water discoloration, which increased with increasing H_2_O_2_ concentrations (Figure 1). Table 1 shows the parameters measured 48 h after H_2_O_2_ addition. The pH values showed no statistically significant differences between H_2_O_2_ treatments in samples stored with light (*p* > 0.05). At the end of treatment, the pH values ranged from 8.88 to 9.22. However, in samples treated with different concentrations of H_2_O_2_ and without light, the pH values decreased one and two units with respect to the initial values (9.47). The final pH ranged from 7.44 to 8.20. This is to be expected since photosynthetic activity was inhibited without light. The magnitude of the pH change is dependent on the intensity of the respiration and photosynthetic activity.

The Optical Density decreased during the assay, from 0.37 (t = 0) to values ranging from 0.08 to 0.09 at the end of the experiment (48 h) (Table 1). No statistically significant differences (*p* > 0.05) between treatments, nor with or without light, were observed.

Dissolved total phosphorus increased at the end of the experiments (Table 1) (*p* < 0.05). These increases most likely occurred as a result of the release of phosphorus during the autolysis process or bacterial lysis [27,28].

The organic matter present may react with H_2_O_2_ and thus degrade it [29]. In the present work, all concentrations of H_2_O_2_ added were rapidly degraded to below the detection limit (0.1 mg·L^−1^) after 48 h, both with and without light. This is in accordance with previous findings that the degradation of natural levels of H_2_O_2_ occurs between 6 and 24 h [30].

#### 2.3.1. Effect of H_2_O_2_ and Light on Chemical Oxygen Demand (COD)

Various authors have reported that organic substances play an important role in stimulating algal blooms, and COD is the most influential factor contributing to changes in the algal blooms [18]. COD represents the loading contributed by a mixture of organic and inorganic substances, possibly including total phosphorous and total nitrogen. In this work, we evaluated the kinetics of COD degradation on addition of various H_2_O_2_ concentrations with and without light. Several kinetics were tested, and the pseudo-first-order kinetic was found to be the most appropriate (Equations (3) and (4) in Material and Methods Section).

The value of (Ko)_COD_ was estimated through the representation of Ln[C/Co] as a function of time, using a linear regression model. The proposed kinetics were successfully applied to the experimental data. Table 2 shows the findings of (Ko)_COD_ values and determination coefficients.

(Ko)_COD_ values were found to be greater as the H_2_O_2_ concentration increased and more pronounced under light, with higher (Ko)_COD_ values (Table 2).

In the range of H_2_O_2_ studied, the times to reach half of the initial COD values were three to five times shorter in light than without light. The (t_m_)_COD_ ranged from 142 to 36 h for different concentrations of H_2_O_2_ under light conditions and from 728 to 126 h in experiments performed without light; 925 h (with light) and 1050 h (without light) were obtained in the control samples (Table 2). These results indicate that H_2_O_2_ addition decreased COD and that the removal is dependent on light.

The greatest (Ko)_COD_ was 1.90 × 10^−2^ h^−1^, which corresponds to the highest concentration applied (3.33 mg·L^−1^) with light. The (t_m_)_COD_ values were 36 and 126 h in experiments with and without light, respectively. The degradation of hydrogen peroxide might lead to differences in the decay of the COD values as they might not follow a pseudo first order decay in this time range.

**Figure 2:**
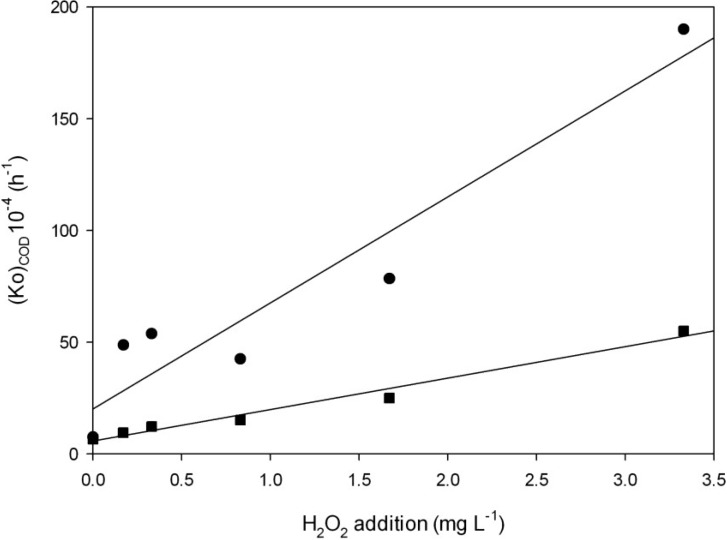
The effects of H_2_O_2_ addition on (Ko)_COD_ ● with light ■ without light. Fitting parameters of Equation (6) were a = 0.0032 ± 0.001, b = 0.005 ± 0.0007 and *R*^2^ = 0.92 for the experiments performed with light; and a = 5.71 ± 1.63, b = 14.07 ± 1.04 and *R*^2^ = 0.98 for experiments without light.

In order to take into account the effects of H_2_O_2_ addition on (Ko)_COD_, Equation (6) was proposed. Figure 2 shows the variation of (Ko)_COD_ as a function of different concentrations of H_2_O_2_ and presence or absence of light. (Ko)_COD_ values increased when the concentration of H_2_O_2_ increased with a good experimental correlation (*R*^2^ = 0.91–0.97) in experiments performed with and without light. (Ko)_COD_ values were almost unrelated to the H_2_O_2_ added and light.

**Table 2:** The parameters obtained by fitting the pseudo-first-order COD and Chl-a kinetics with experimental data. Ko: pseudo first order decay constant (h^−1^); (t_m_)_COD_: time required to the concentration of COD decay to half initial values (h); (t_m_)_Chl-a_: time required for the concentration of Chl-a to decay to half its initial value (h). Different letters in each column indicate significant differences.

**Chemical oxygen demand (COD) Decay**						
H_2_O_2_ addition	With light			Without light		
	(Ko)_COD_ (h)^−1^	*R*^2^	(t_m_)_COD_ (h)	(Ko)_COD_ (h)^−1^	*R* ^2^	(t_m_)_COD_ (h)
0	7.5 × 10^−4^ ± 5.1 × 10^−4^	0.55	925 ± 629 ^a^	6.5 × 10^−4^ ± 1.2 × 10^−4^	0.86	1050 ± 194 ^a^
0.17 mg·L^−1^	4.9 × 10^−3^ ± 1.5 × 10^−3^	0.70	142 ± 43 ^b^	9.5 × 10^−4^ ± 3.8 × 10^−4^	0.55	728 ± 291 ^a^
0.33 mg·L^−1^	5.4 × 10^−3^ ± 1.0 × 10^−3^	0.56	128 ± 24 ^b^	1.3 × 10^−3^ ± 6.3 × 10^−4^	0.45	533 ± 258 ^a^
0.83 mg·L^−1^	4.3 × 10^−3^ ± 9.5 × 10^−4^	0.87	163 ± 36 ^b^	1.5 × 10^−3^ ± 1.5 × 10^−4^	0.44	459 ± 46 ^b^
1.67 mg·L^−1^	7.8 × 10^−3^ ± 4.9 × 10^−3^	0.70	88 ± 55 ^b^	2.6 × 10^−3^ ± 7.5 × 10^−4^	0.34	277 ± 80 ^b^
3.33 mg·L^−1^	1.9 × 10^−2^ ± 9.9 × 10^−3^	0.85	36 ± 18 ^b^	5.6 × 10^−3^ ± 2.5 × 10^−3^	0.50	126 ± 56c ^c^
**Chlorophyll-a (Chl-a) Decay**						
H_2_O_2_ addition	With light			Without light		
	(Ko)_Chl-a_ (h)^−1^	*R* ^2^	(t_m_)_Chl-a_ (h)	(Ko)_Chl-a_ (h)^−1^	*R* ^2^	(t_m_)_Chl-a_ (h)
0	0.010 ± 0.002	0.84	69 ± 13.8 ^a^	0.011 ± 0.002	0.86	63 ± 11.4 ^a^
1.67 mg·L^−1^	0.057 ± 0.008	0.89	12 ± 1.7 ^b^	0.050 ± 0.009	0.79	14 ± 2.5 ^b^
0.17 mg·L^−1^	0.042 ± 0.008	0.86	16 ± 3.1 ^b^	0.025 ± 0.008	0.86	28 ± 8.8 ^b^
0.33 mg·L^−1^	0.050 ± 0.009	0.83	14 ± 2.5 ^b^	0.041 ± 0.008	0.87	17 ± 3.3 ^b^
0.83 mg·L^−1^	0.050 ± 0.008	0.87	14 ± 2.2 ^b^	0.045 ± 0.008	0.85	15 ± 2.6 ^b^
3.33 mg·L^−1^	0.060 ± 0.009	0.85	12 ± 1.8 ^b^	0.051 ± 0.008	0.79	14 ± 2.1 ^b^

Note: ^a, b, c,^ indicate significant differences between treatments at the 5% level of probability (*p* < 0.05).

The parameters of Equation (6) allow us to predict the (Ko)_COD_ and then use Equation (4) to estimate the COD values at times and concentrations of H_2_O_2_ unlike those applied in the present work in the range of H_2_O_2_ studied. Thus, it is possible to predict the COD values after 48 h by knowing the initial values of COD. This indicates that H_2_O_2_ is effective in lowering the COD and this effect is enhanced by the presence of light. Applying H_2_O_2_ under field conditions could reduce COD values and decrease the likelihood of blooms. COD is a synthetic indicator of water contamination representing the degree of organic pollution in water [31]. High COD levels in water are toxic and can affect the aquatic environment. Furthermore, the increase in COD can be generated by the degradation of phytoplankton blooms [32]. Thus, the application of H_2_O_2_ could help improve the water quality of polluted aquatic systems by reducing COD, coliforms and the algae biomass by Chl-a degradation (see Section 2.3.2).

#### 2.3.2. Effect of H_2_O_2_ and Light on Chl-a Degradation

We evaluated the effect of adding H_2_O_2_ on Chl-a degradation. Before H_2_O_2_ application, the Chl-a content was 530 μg·L^−1^. In the control samples, the Chl-a contents were 446 and 420 μg·L^−1^ at 48 h of the initial experiment performed under light and without light, respectively (Table 1). The decrease in Chl-a values in the control samples could indicate that the cultures were slightly stressed (e.g., culture conditions led to a change in the diurnal rhythm as they were performed under light). In view of this, photoautotrophs could have been slightly more sensitive to the hydrogen peroxide than under optimal conditions.

After 48 h, Chl-a concentration was found to be five to ten times lower than before H_2_O_2_ was added, depending on the concentration of H_2_O_2_ added and the light conditions. In the experiments performed with light and with 0.17, 0.33, 0.83, 1.67 and 3.33 mg·L^−1^ of H_2_O_2_, the Chl-a content decreased by 78%, 86%, 86%, 86% and 89% respectively after 48 h. In experiments performed without light, Chl-a decreased 40%, 86%, 86%, 78% and 79%, respectively.

Our results are consistent with those of Barroin and Feuillade [33], who showed that a low concentration of hydrogen peroxide destroys Chl-a and also other cyanobacterial pigments. Randhawa [34] informed that Chl-a in cyanobacterial cultures treated with 1.6, 3.2 and 6.4 mg·L^−1^ H_2_O_2_ decreased more than 90% in 24 h. Qian *et al.* [35] found that cyanobacterial Chl-a decreased to 13.9% below the control after 48 h of 1.3 mg·L^−1^ H_2_O_2_ exposure under light in *Microcystis aeruginosa* cultures.

**Figure 3:**
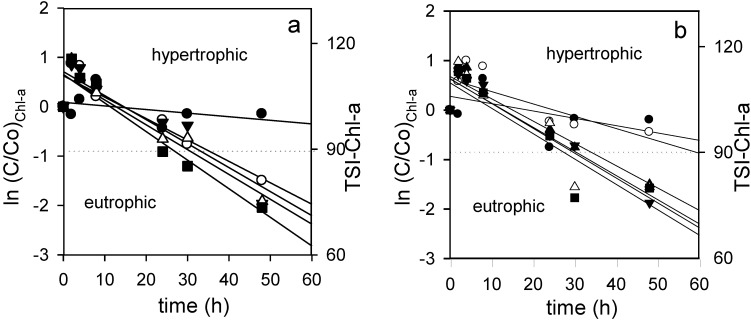
Chlorophyll-a (Chl-a) decay upon application of different initial concentration of H_2_O_2_ ● control; ○ 0.17 mg·L^−1^; ▼ 0.33 mg·L^−1^; ▲ 0.83 mg·L^−1^; ∆ 1.67 mg·L^−1^; ■ 3.33 mg·L^−1^; (**a**) with light; (**b**) without light.

Figure 3a,b shows the natural logarithm of cyanobacterial Chl-a decay concentration obtained in the experiments as a function of time under the tested conditions. TSI values were included in the figure to visualize changes in the trophic state. It can be observed that the Chl-a decay rate increases as a function of H_2_O_2_ addition. We applied the pseudo-first-order kinetic in Equation (7) that was found to be the most appropriate. Table 2 shows the findings of (Ko)_Chl-a_ values and coefficients of determination. (Ko)_Chl-a_ values increased as the H_2_O_2_ concentration increased. Chl-a decay was more pronounced under light, with higher (Ko)_Chl-a_ values (Table 2). Similarly, the times to reach half of the initial Chl-a concentration (t_m_)_Chl-a_ were calculated using (Ko)_Chl-a_ values from Table 2.

The (t_m_)_Chl-a_ ranged between 12 and 6 h for different concentrations of H_2_O_2_ under light and 14–28 h in experiments performed without light. For control samples, (t_m_)_Chl-a_ values were 69 h (under light) and 63 h (darkness) (Table 2).

We found that 0.33 mg·L^−1^ of H_2_O_2_ was the lowest effective concentration to reduce the Chl-a concentration by half in 14 and 17 h under light and darkness, respectively; no statistically significant differences were found at higher concentrations of H_2_O_2_ (Table 2). Such concentration overlaps with the natural levels of 0.34 mg·L^−1^ observed in freshwater [35,36].

The greatest (Ko)_Chl-a_ was 0.060 h^−1^, which corresponds to the highest concentration applied (3.33 mg·L^−1^) in experiments performed with light. In this condition, the time required for Chl-a decay to half of the initial values was 12 and 14 h under light and darkness, respectively.

The first order rate constants for Chl-a decay (Ko)_Chl-a_ exhibited a sigmoidal regression, indicating that it is likely that there is a H_2_O_2_ concentration below which Chl-a decay is negligible, and a concentration above which the decay rate does not increase any further.

In order to take into account the effects of H_2_O_2_ addition on (Ko)_Chl-a_, Equation (8) was proposed. The fitting parameters were a = 0.055 ± 0.003, b = 7.93 ± 2.63 and *R*^2^ = 0.99 and a = 0.049 ± 0.003, b = 4.54 ± 2.09 and *R*^2^ = 0.99 in experiments performed with and without light, respectively. These parameters allow us to calculate (Ko)_Chl-a_ values at H_2_O_2_ concentrations different from those which were applied in the present work in the range of H_2_O_2_ studied. Thus, it is possible to predict the Chl-a values after 48 h by knowing the initial values of Chl-a.

**Figure 4:**
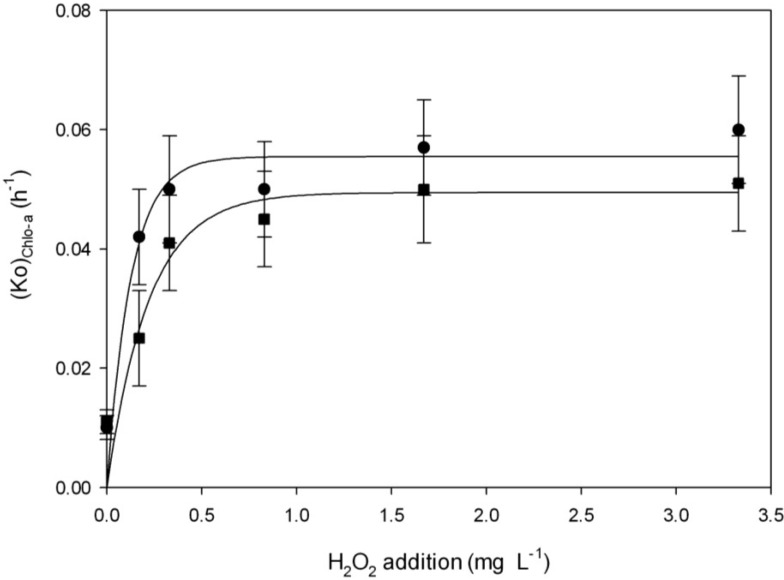
The effect of H_2_O_2_ addition on Chl-a decay (Ko)_Chl-a_ ● with light ■ without light.

Figure 4 illustrates the variation in (Ko)_Chl-a_ as a function of H_2_O_2_ concentration and the presence or absence of light, showing that (Ko)_Chl-a_ values exponentially increase when the concentration of H_2_O_2_ rises, with a good experimental correlation (*R*^2^ = 0.99).

These tests were conducted during a period of up to 48 h under laboratory conditions. It is possible that in aquatic environments, Chl-a levels could increase from this time due to the proliferation of phytoplankton.

### 2.4. The Effect of H_2_O_2_ and Light on Phytoplankton Counts

**Figure 5:**
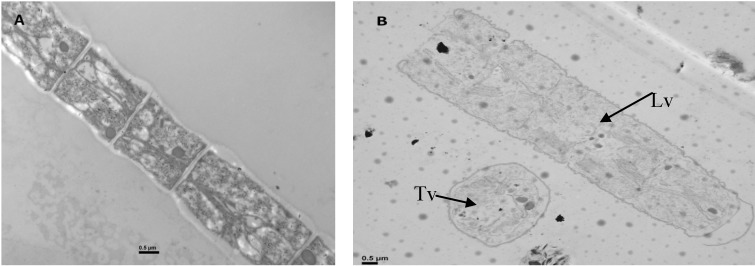
(**A**) Transverse view of the trichome of *Planktothrix agardhii* (control); (**B**) Transverse (Tv) and longitudinal (Lv) view of *P. agardhii* trichome under 3.33 mg·L^−1^ of H_2_O_2_, after 48 h of treatment with light.

The effects of different concentrations of H_2_O_2_ on phytoplankton counts were analyzed during the experiment in both light and darkness. Before the assay, cyanobacteria predominated (2.4 × 10^7^ cells mL^−1^) while other phytoplankton groups remained below 1% of total phytoplankton (4.0 × 10^3^ cells mL^−1^). Forty-eight hours after adding H_2_O_2_, cyanobacteria still dominated and cell density did not change significantly in respect to the initial values (*p* > 0.05). None of the experiments showed a significant decrease in cell density after 48 h (data not show). Although the density did not change, electron microscopic observations of *Planktothrix agardhii* showed filaments and cells in several stages of degeneration, effects that were more severe under higher H_2_O_2_ concentrations and longer time of exposure. Swollen cell walls and some degree of cytoplasmatic alteration were observed after 24 h of treatment with 0.83 mg·L^−1^. After 48 h under the highest concentration (3.33 mg·L^−1^), we observed cell wall and plasmatic membrane disruption, disorganized and degraded thylakoids and changes in the cytoplasmatic inclusions (Figure 5).

Similar results were found by Matthijs *et al.* [13], who reported a rapid decline of photosynthetic vitality in field material dominated by *Plankthotrix agardhii* after incubation with different concentrations of H_2_O_2_. The cyanobacterial population collapsed within a few days and stayed low for seven weeks, while the remaining plankton community appeared much less affected. Levels of 1, 2 and 4 mg·L^−1^ resulted in the suppression of the photosynthetic vitality to less than 30% of the control within 3 h.

It has been proposed that H_2_O_2_ may attack the cyanobacteria cells by forming hydroxyl and hydroperoxyl radicals that inhibit the photosynthesis activity by blocking photosynthetic electron transfer [9]. As in this study, Drábková *et al.* [10] reported that H_2_O_2_ toxicity under light condition was increased, most likely by an enhanced conversion to the hydroxyl radicals (OH*) that are known to be the strongest reactive oxygen species (ROS).

Samuilov *et al.* [37] reported that H_2_O_2_ inhibits photosynthetic O_2_ evolution, because hydroxyl and hydroperoxyl radicals can lead to the inactivation of photosystem II, destroy pigment synthesis and the integrity of membrane, and result in cyanobacterial cell death. In the present work, no differences were found in the cells numbers of phytoplankton species to H_2_O_2_ addition since cyanobacteria was the predominant group in the shallow lake samples. Chlorophyta and chrysophyta represented less than 1% of total phytoplankton, quantities that did not change during the experiment. However, it is important to mention that the more sensitive parameter, TEM, showed that *Planktothrix agardhii* was damaged.

A selective effect of H_2_O_2_ on the phytoplankton community was reported by Drábková *et al.* [10]. Cyanobacteria was affected by H_2_O_2_ at 10 times lower concentrations than green alga and diatoms, and a strong light dependent toxicity enhanced the difference. Single concentrations of 0.27 mg·L^−1^ of H_2_O_2_ caused 50% inhibition to *M. aeruginosa* at high irradiance. The higher rate of H_2_O_2_ decomposition leads to stronger oxidizing and cyanobacterial effects [10]. The inhibition of photosynthesis was more severe in five tested cyanobacterial species than in three green algal species and one diatom species. Hence the inhibitory effect of H_2_O_2_ is especially pronounced for cyanobacteria [8], showing that hydrogen peroxide is a compound selective to cyanobacteria.

There are several reasons to justify the selectivity observed: phycobilisomes of cyanobacteria are situated on the outside of the thylakoid membranes directly exposed to the cytoplasm rather than in organised chloroplasts, and this structure makes the photosynthetic apparatus more readily susceptible to external H_2_O_2_ than in green alga or diatoms with photosystems enclosed in chloroplasts [6]. Furthermore, cyanobacteria have less elaborate detoxification enzymes such as catalase or catalase-peroxidase. The light inactivation of catalase [38,39] makes cyanobacteria even more susceptible to H_2_O_2_ in high light intensities.

The intracellular toxicity mechanisms of hydrogen peroxide in *Microcystis aeruginosa*, were studied by Mikula *et al.* [40], who reported that light is one of the critical factors affecting H_2_O_2_ decomposition and thus greatly influences its toxicity. Using applied flow cytometry and Chl-a fluorescence measurements, the authors suggested that hydrogen peroxide exposure elicits an immediate decline of metabolic (esterase) activity, and immediate changes in Chl-a fluorescence parameters, followed by an increase in the percentage of membrane-compromised cells.

The results of the present work show that the increase in H_2_O_2_ destroyed Chl-a, decreased COD, and damaged the membrane integrity of *Planktothrix agardhii* in laboratory experiments. These effects are exaggerated in the presence of light. In the same way, total and faecal coliforms were inhibited with H_2_O_2_ addition, and this effect was enhanced by light, suggesting that the generation of ROS could be the principal mechanism. The release of stored phosphorus during the autolysis process or bacterial lysis could explain the increase in dissolved phosphorus values. This effect was stronger in the presence of light.

Our results demonstrate that H_2_O_2_ could be potentially used in hypertrophic systems because it not only collapses cyanobacterial cells but may also reduce Chl-a content and chemical oxygen demand. It may also be possible to use H_2_O_2_ to remove cyanobacteria from shallow lakes. We propose the application of H_2_O_2_ when the cyanobacterial bloom starts and under higher light conditions. According to Matthijs [13], 2 mg·L^−1^ of H_2_O_2_ could be applied homogeneously into the entire water volume of the lake in the morning or evening to achieve more effective action. However, further investigation into the effects of H_2_O_2_ addition on ecosystem function must be conducted before this could be implemented. In addition, the release and increase of dissolved total phosphorus poses a problem because it can contribute to the eutrophication of the water body. The impact on elements of the ecosystem such as larval fish and macro-invertebrates and zooplankton should be assessed.

## 3. Experimental Methodology

### 3.1. Sample Collection

Sampling was carried out on December 2010 in the shallow lake of Los Patos, a small (surface area 2.5 ha, maximum depth 1 m) and hypertrophic freshwater body located in Ensenada city, 60 km south–west of the city of Buenos Aires, Argentina. Intense cyanobacterial blooms dominated by *Planktothrix agardhii* and *Raphiodiopsis mediterranea* have been frequently observed during the past decades as the result of nutrient overloading. This shallow lake contributes to the region as a valuable landscape element and is also used for recreational activities and fishing.

Water samples were taken 40 cm below the water surface at one particular point (34°50'44'' S, 57°57'26'' W) using a van Dorn bottle. In total, 200 mL of water were subsampled into a polyethylene bottle and preserved in Lugol’s iodine solution for phytoplankton enumeration. Surface water temperature, pH and conductivity were measured *in situ* using a Sonda Sper Scientific Water Quality Meter 850081. The total orthophosphate phosphorus, dissolved total phosphorus, chemical oxygen demand (COD), Optical Density (OD), Chl-a, faecal coliforms and total coliforms were measured once samples arrived at the laboratory (see Analyzed parameters and analytical methods).

Sample collection was performed according to the GEMS/Water Operational Guide [41].

### 3.2. Laboratory Incubations and Experimental Design

Samples taken from Los Patos shallow lake were subdivided into 1 L aliquots that were put in 1.5 L incubation bottles.

Drábková *et al.* [10] and Matthijs *et al.* [13] showed that cyanobacteria are sensitive even to low amounts of mg·L^−1^ of H_2_O_2_. Accordingly, we added different volumes of a 30 g·L^−1^ (3% w/v) stock solution of H_2_O_2_ (Fluka), resulting in final concentrations of 0, 0.17, 0.33, 0.83, 1.67 and 3.33 mg·L^−1^.

After adding H_2_O_2_, the bottles were stirred for 30 s. Bottles without H_2_O_2_ added were used as a control. Three replicates for each concentration were conducted.

The H_2_O_2_ experiments were carried out in the incubation cabinet without aeration or shaking during the incubation and stored at room temperature (24 ± 1 °C) under two experimental conditions: continuous irradiance of 50 μmol photon m^−2^·s^−1^, provided by white light fluorescent tubes, and continuous darkness. Light intensity was measured in the lab using an Underwater Quantum Sensor (LI-192 US-SQS; LI-COR, Lincoln, NE, USA) and a LI-250A Light Meter (LI-COR).

Samples for Chl-a, physic-chemical parameters, faecal and total coliforms were determined at the beginning (t = 0) and at 2, 4, 8, 24, 30 and 48 h after 0.17, 0.33, 0.83, 1.67 and 3.33 mg·L^−1^ H_2_O_2_ addition.

At the end of the experiment (48 h) we analyzed H_2_O_2_ concentration by peroxide Quantofix test sticks (Machereye-Merck, Darmstadt, Germany). We were able to refine the measurements by taking photographs of each test stick and subsequent comparison to our own calibration series according to the procedure described by Matthijs *et al.* [13].

### 3.3. Analyzed Parameters and Analytical Methods

Standard analytical methods developed and/or compiled by the American Public Health Association [42] were used for each chosen parameter (the method number for each determination is provided between parentheses): pH (4500H-B), temperature (2550B), conductivity (2510A), Optical Density (OD)—measured using a Metrolab Spectrophotometer (Metrolab, Argentina) at 740 nm.

The orthophosphate phosphorus levels were determined over samples previously treated by acid hydrolysis and digested with persulphate (4500-P); dissolved total phosphorus was determined previously filtered through 0.45 μm cellulose acetate membranes (GE Osmonics).

Chemical Oxygen Demand (COD) was determined by potassium dichromate in 50% sulfuric acid solution at reflux temperature (5220D), Chl-a (10200H-spectrophotometric) with 90% buffered acetone), faecal coliforms (9221 E) and total coliforms were estimated using the multiple-tube fermentation test (9221B) and the results were expressed as Most Probable Number (MPN × 100 mL^−1^).

Aliquots of 10 mL of the control and two H_2_O_2_ treatments (0.833 mg·L^−1^ and 3.333 mg·L^−1^) were preserved in Lugol’s iodine solution for phytoplankton counting and immediately quenched with sodium thiosulphate. Phytoplankton was taxonomically identified using methodology described by Komàrek and Anagnostidis [43] and quantifications were done with an inverted microscope (AXIOVERT 40 C, Carl Zeiss, Jena, Germany) using the Utermöhl method [44].

#### Trophic State Index

The Trophic State Index (TSI) of Carlson [45] as modified by Aizaki [46] was applied to the shallow lake and calculated using the following formula:

(1) TSI- Pt=10×[2.46+(6.68+1.15 ln Pt*)ln 2.5]

_t_P = Total Phosphorus (mg·L^−1^)

(2) $$\text{TSI- Chl-a}=\text{10 }\times (\text{2.46}+\frac{\text{ln Chl-a**}}{\text{ln 2.5}})$$

Based on the values of TSI, water bodies are classified as Oligotrophic (TSI < 30), Mesotrophic (30 < TSI ≤ 60), Eutrophic (60 < TSI < 90) and Hypertrophic (TSI > 90).

### 3.4. Electron Microscopy Studies

Screening tests to evaluate morphological changes were studied with a JEOL JEM 1200–EX II Transmission Electron Microscope (TEM) (JEOL, Peabody, MA, USA), and images were captured using a digital camera Erlangshen ES 1000 W. We analyzed the light samples 48 h after 3.33 mg·L^−1^ H_2_O_2_ was added. For TEM preparations, cells were collected by centrifuging at 1500 rpm for 10 min. The samples were fixed in 2.0% glutaraldehyde at 4 °C for 2 h and then rinsed in phosphate buffer. Next, they were post fixed in 1% osmic acid for 2 h at 4 °C and rinsed in phosphate buffer. After that, the samples were dehydrated in increasing concentrations of alcohol (50%, 70%, 90%, and 100%, 10 min in each) and 100% acetone. The material was embedded in Epon 812 overnight at 60 °C. Using a Reichert-J Super Nova Ultracut, the Epon blocks were cut into ultrathin sections (60 nm), which were contrasted with urany acetate and lead citrate [47].

### 3.5. Modelling COD and Chl-a Decay

Several kinetics were tested for modelling COD decay in terms of time. The pseudo-first-order kinetic was found to be the most appropriate:

(3) $$\frac{dC}{dt}=-{\left(Ko\right)}_{COD}\times C$$

Integrating $\int \frac{dC}{C}=-{\left(Ko\right)}_{COD}.\int t$

(4) $$Ln\left[\frac{C_{COD}}{C_{\text{0 }COD}}\right]=-{\left(Ko\right)}_{COD}\times t$$

where (*Ko*)*_COD_* = COD pseudo first order decay constant (h^−1^); *C_COD_* = COD content at time (*t*) (mg·L^−1^); *C*_0*COD*_ = initial COD content (mg·L^−1^); *t* = time (h). 

When the COD is COD/2, the time to reach half of the initial COD values (*t_m_*)*_COD_* was calculated as:

(5) $${\left(t_{m}\right)}_{COD}=-\frac{\ln2}{{\left(Ko\right)}_{COD}}$$

In order to take into account the effects of H_2_O_2_ addition on (*Ko*)*_COD_*, the following Equation was proposed

(6) $${\left(Ko\right)}_{COD}=a+b\times C$$

where *a* is the interception of y axes and *b* is the slope and *C* is the concentration of H_2_O_2_ used.

For chlorophyll decay we applied the pseudo-first-order kinetic in Equation (7) and found it to be the most appropriate:

(7) $$Ln\left[\frac{C_{Chl-a}}{C_{\text{0 }Chl-a}}\right]=-{\left(ko\right)}_{Chl-a}\times t$$

where (*Ko*)*_Chl-a_* = pseudo first order Chl-a decay constant (h^−1^); *C_Chl-a_* = Chl-a concentration at time (*t*); *C*_0*Chl-a*_ = initial Chl-a concentration (mg·L^−1^); *t* = time (h)

The relationship between (*Ko*)*_Chl-a_* and concentration of H_2_O_2_ addition was modelled by

(8) $${\left(Ko\right)}_{Chl-a}=a(1-e^{-b.c})$$

where *a* and *b* are empirical constants and *c* is the concentration of H_2_O_2_ added.

### 3.6. Statistical Analysis

All the experiments were performed in triplicate. The Analysis of Variance (ANOVA) and the test of mean comparison according to Fisher LSD were applied with significance levels of 0.05, using a statistical package for computers (SYSTAT Inc. 2007, version 12.0, Chicago, IL, USA). The statistical requirements for the ANOVA (normal distribution, homogeneity of variance) were performed.

The parameters tested were: conductivity, pH, optical density, total and dissolved phosphorous content, phytoplankton counts, COD and Chl-a content, obtained from treated samples and the initial values.

## 4. Conclusions

In our laboratory assay of natural water samples from lake Los Patos, the addition of H_2_O_2_ reduced the Chl-a content as well as the COD by more than 90% in a few hours, depending on the experimental concentration.

Modelling COD and Chl-a decay has allowed us to calculate (Ko)_COD_ and (Ko)_Chl-a_ values at H_2_O_2_ concentrations different from those that were applied in the present work in the range of H_2_O_2_ studied. Thus, it is possible to predict the COD and Chl-a values after 48 h by knowing the start values of COD and Chl-a.

A concentration of 0.33 mg·L^−1^ of H_2_O_2_ was the lowest effective concentration for half decay in Chl-a content at 14 and 17 h, with light and without light, respectively, whereas 3.33 mg·L^−1^ of H_2_O_2_ addition reduced the COD values to half at 36 and 126 h in experiments under light and darkness, respectively. Hence, the presence of light enhanced the effects of H_2_O_2_ addition.

Although the densities of the dominating cyanobacterial cells did not decrease under H_2_O_2_, TEM observations of the trichomes of the dominating *Planktothrix agardhii* showed several stages of degeneration and cell damage 48 h after 3.33 mg·L^−1^ of H_2_O_2_ was added in the presence of light. The relatively short period of 48 h might be the reason for missing any difference in the sensitivity of different taxa of phytoplankton, as this time was too short to cause a shift in the phytoplankton community.

In accordance with the observed cell damage (destroyed pigment and membrane integrity) in cyanobacteria, the total and faecal coliforms were also completely inhibited 48 h after adding 3.33 mg·L^−1^ of H_2_O_2_.

Our results indicate that H_2_O_2_ could be implemented to treat highly polluted systems and develop strategies for water quality management, since it can contribute to a reduction in Chl-a, coliform bacteria and COD.
